# Allergen Microarray Indicates Pooideae Sensitization in Brazilian Grass Pollen Allergic Patients

**DOI:** 10.1371/journal.pone.0128402

**Published:** 2015-06-11

**Authors:** Priscila Ferreira de Sousa Moreira, Katharina Gangl, Francisco de Assis Machado Vieira, Leandro Hideki Ynoue, Birgit Linhart, Sabine Flicker, Helmut Fiebig, Ines Swoboda, Margarete Focke-Tejkl, Ernesto Akio Taketomi, Rudolf Valenta, Verena Niederberger

**Affiliations:** 1 Laboratory of Allergy and Clinical Immunology, Biomedical Science Institute, Federal University of Uberlândia, Uberlândia, Minas Gerais, Brazil; 2 Department of Otorhinolaryngology, Medical University of Vienna, Vienna, Austria; 3 Christian Doppler Laboratory for Allergy Research, Division of Immunopathology, Department of Pathophysiology and Allergy Research, Center for Pathophysiology, Infectiology and Immunology, Medical University of Vienna, Vienna, Austria; 4 Department of Medicine, University of Caxias do Sul, Rio Grande do Sul, Brazil; 5 Division of Immunopathology, Department of Pathophysiology and Allergy Research, Center for Pathophysiology, Infectiology and Immunology, Medical University of Vienna, Vienna, Austria; 6 Allergopharma J. Ganzer KG, Reinbek, Germany; French National Centre for Scientific Research, FRANCE

## Abstract

**Background:**

Grass pollen, in particular from *Lolium multiflorum* is a major allergen source in temperate climate zones of Southern Brazil. The IgE sensitization profile of Brazilian grass pollen allergic patients to individual allergen molecules has not been analyzed yet.

**Objective:**

To analyze the IgE sensitization profile of a Brazilian grass pollen allergic population using individual allergen molecules.

**Methods:**

We analyzed sera from 78 grass pollen allergic patients for the presence of IgE antibodies specific for 103 purified micro-arrayed natural and recombinant allergens by chip technology. IgE-ELISA inhibition experiments with *Lolium multiflorum*, *Phleum pratense* extracts and a recombinant fusion protein consisting of Phl p 1, Phl p 2, Phl p 5 and Phl p 6 were performed to investigate cross-reactivities.

**Results:**

Within the Brazilian grass pollen allergic patients, the most frequently recognized allergens were Phl p 1 (95%), Phl p 5 (82%), Phl p 2 (76%) followed by Phl p 4 (64%), Phl p 6 (45%), Phl p 11 (18%) and Phl p 12 (18%). Most patients were sensitized only to grass pollen allergens but not to allergens from other sources. A high degree of IgE cross-reactivity between *Phleum pratense*, *Lolium multiflorum* and the recombinant timothy grass fusion protein was found.

**Conclusions:**

Component-resolved analysis of sera from Brazilian grass pollen allergic patients reveals an IgE recognition profile compatible with a typical Pooideae sensitization. The high degree of cross-reactivity between *Phleum pratense* and *Lolium multiflorum* allergens suggests that diagnosis and immunotherapy can be achieved with timothy grass pollen allergens in the studied population.

## Introduction

Grass pollen allergens are considered to be the most important cause of seasonal allergy worldwide. In some areas, the level of sensitization is about 20% of the general population and 40% of atopic individuals. [1] Prevalence of grass pollen allergy varies depending on climate, environmental factors such as degree of exposure, air pollution as well as genetic predisposition of the subject [2–4].

It has been shown that most clinically relevant grasses belong to the Pooideae subfamily, although in certain climatic and geographic areas such as the Mediterranean area and areas with subtropical climate, grasses from other families, such as Bermuda grass (*Cynodon dactylon;* subfamily: Chloridoideae), may also play an important role. [5, 6] In the subtropical high altitude zones of Brazil, *Lolium multiflorum* (Italian or annual ryegrass; subfamily: Pooideae) has been shown to be a major sensitizing pollen source in patients with grass pollen allergy. [7–9] However, also other grass species, such as *Anthoxanthum odoratum* (sweet vernal grass; subfamily: Pooideae), *Cynodon dactylon* (Bermuda grass; subfamily: Chloridoideae), *Holcus lanatus* (common velvet grass; subfamily: Pooideae), *Paspalum notatum* (bahia grass; subfamily: Panicoideae) and *Bromus* sp (subfamily: Pooideae) occur there. Cross-reactivity studies with single and mixed natural grass pollen extracts have demonstrated a certain level of cross-reactivity between *Lolium multiflorum* and other grasses in this region, but the clinical relevance of this finding has not yet been investigated using recombinant allergens. [8–10].

During the last 20 years several hundred different allergens have been produced as recombinant allergens, and component-resolved diagnosis has been used to analyze allergic patients’ sensitization profiles. [11, 12] Using recombinant grass pollen allergens it has become possible to diagnose and treat grass pollen allergy. [13–16] Moreover, recombinant grass pollen allergens allow to dissect sensitization profiles which are indicative of a sensitization to major grass pollen allergy subfamilies such as the Pooideae and Chloridoideae. Recombinant allergen-based diagnosis may therefore be useful to identify the culprit grass pollen allergen sources and to select appropriate allergens for specific immunotherapy (SIT). [17]

Here we analyzed the allergen profile recognized by grass pollen allergic patients from Brazil using 103 micro-arrayed purified allergen molecules using the Immuno Solid-phase Allergen Chip (ISAC). Our results revealed a predominant Pooideae-type of sensitization. We then investigated the level of cross-reactivity between timothy grass, Italian rye grass pollen extract and a recombinant fusion protein consisting of the four major timothy grass pollen allergens (Phl p 1, Phl p 2, Phl p 5 and Phl p 6) to evaluate the potential usefulness of recombinant timothy grass pollen allergens for diagnosis and treatment in the temperate climate zones of Brazil. [18]

## Methods

### Patients

Sera from seventy-eight grass pollen allergic patients were analyzed. Subjects represented consecutive patients who attended the allergy clinic of Dr. Francisco Vieira, in Caxias do Sul, Southern Brazil, which treats allergic patients suffering from allergic asthma and allergic rhinitis. Patients were routinely skin prick tested with the following panel of inhalant allergens: Grasses (mix); *Dermatophagoides pteronyssinus*, *Dermatophagoides farinae*, *Blomia tropicalis*, and *Lolium multiflorum* during their first assessment in the allergy clinic. All study patients were selected according to a positive case history indicative of seasonal allergic rhinitis, allergic conjunctivitis and/or allergic asthma during the grass pollen season and positive skin prick tests (wheal diameter ≥ 3mm) to grass pollen (*Lolium multiflorum* skin prick test (= SPT) 100% positive, Grasses (mix) SPT 100% positive). The grass pollen season in Caxias do Sul shows peak grass pollen counts in November, ranging from 512 to 949 grains per m^3^/air [19].

Prevalence of mite sensitivity among the seventy-eight patients according to the SPTs was *Dermatophagoides pteronyssinus*:45%, *Dermatophagoides farinae*: 27%, *Blomia tropicalis*: 29%. None of the subjects had received immunotherapy against grass pollen allergens. After written informed consent had been given, serum samples were collected and skin prick tests were performed in each subject. Serum samples were stored at -20°C until use.

The study was approved by the Ethical Committee in Human Research at the Federal University of Uberlândia and anonymized serum samples were analyzed with approval of the Ethics Committee of the Medical University of Vienna. A summary of the demographic and clinical data from the 78 grass pollen allergic patients is shown in Table 1.

**Table 1:** Demographic and clinical characterization of the grass pollen allergic patients.

**Demographic Characteristics**	Number of subjects	78
	Mean Age (years)	31 +-10
	Gender (m/f)	28/50
**Clinical Symptoms (n,%)**	Rhinitis	76 (97%)
	Conjunctivitis	65 (83%)
	Asthma	4 (5%)
**Positive SPT (n,%)**	Grass (mix)	78 (100%)
	Lolium multiflorum	78 (100%)
	D. pteronyssinus	35 (45%)
	D. farinae	21 (27%)
	Blomia tropicalis	29 (29%)

SPT: skin prick test

### Grass pollen extracts, recombinant and natural allergens

*Lolium multiflorum* pollen was purchased from Greer Laboratories (Lenoir NC, USA). *Phleum pratense* pollen was purchased from Allergon (Välinge, Sweden). Pollen protein extracts were prepared by homogenizing 1g pollen in 50 ml of distilled water, containing 2mM phenylmethansulfonylfluorid (= PMSF) using an ultraturrax (Ika, Heidelberg Germany) and extraction of the homogenate overnight at 4°C under continuous shaking. Homogenates were centrifuged at 10.000xg for 15 min at 4°C to remove insoluble particles. Supernatants were frozen in liquid nitrogen as 1ml aliquots, lyophilized and stored at -20°C until use. Lyophilized extracts were resuspended in 220μl water and the protein content and the quality of the protein extracts was analyzed by sodium dodecyl sulphate polyacrylamide gel electrophoresis (= SDS-PAGE) and Coomassie blue staining. The protein concentration was determined with the bicinchoninic acid (= BCA) Protein Assay kit (Novagen, Merck Millipore, Billerica, USA).

rPhl p 6251, a fusion protein [16] consisting of the four major timothy grass pollen allergens (Phl p 1, Phl p 2, Phl p 5, Phl p 6), as well as recombinant allergens (rBet v 1, rPhl p 1, rPhl p 2, rPhl p 5, rPhl p 6, rPhl p 12) were obtained from Biomay (Vienna, Austria). Purified natural Phl p 4 was obtained from Allergopharma (Reinbek, Germany). [20]

### Immunoblots

Grass pollen extracts were separated by preparative SDS-PAGE and blotted onto nitrocellulose. [21] Nitrocellulose-blotted allergen extracts were probed with rabbit antibodies specific for grass group 1, 4, 5, 6, 7, 12 or 13 allergens diluted 1:5000 in buffer A [50mM Na phosphate, pH 7.5, 0.5% w/v bovine serum albumin (BSA), 0.5% v/v Tween 20, 0.05% NaN_3_]. The antibodies had been produced by immunization of rabbits with recombinant *Phleum pratense* allergens (Biomay, Vienna, Austria; immunization by Charles River, Kisslegg, Germany). Bound rabbit antibodies were detected using a iodine 125 (= ^125^I)-labeled goat anti-rabbit IgG antibody (Perkin Elmer, USA) diluted 1:3000 in buffer A. Group 2 allergens were detected using a human monoclonal anti-Phl p 2 antibody at a concentration of 0.1μg/mL (mAb2) [22] followed by incubation with a mouse monoclonal anti-human IgG_1_ (BD Biosciences, San Jose, California, USA) and detection with a ^125^ I-labeled goat anti mouse IgG antibody (PerkinElmer, Inc., San Jose, California, USA) diluted 1:500. Bound antibodies were visualized by autoradiography using KODAK X-OMAT films and intensifying screens (Kodak, Heidelberg, Germany).

### IgE reactivity to micro-arrayed allergens—ISAC

Chips containing micro-arrayed natural and recombinant allergens (Immuno-Solid phase Allergen Chip, ISAC, Thermofisher/Phadia, Uppsala, Sweden) were used to detect IgE antibodies specific for 103 different recombinant or purified natural allergens. The version of the ISAC chip used for this study contained 9 different timothy grass pollen allergens (rPhl p 1, rPhl p 2, nPhl p 4, rPhl p 5, rPhl p 6, rPhl p 7, rPhl p 11, rPhl p 12) and the Bermuda grass pollen allergen (nCyn d 1). The allergen repertoire of the used ISAC chip is detailed in Table 2. The assay was performed according to the manufacturer´s instructions.

**Table 2:** Allergen repertoire of the ISAC microarray.

No.	Allergen	No.	Allergen	No.	Allergen
1	Act d 1	36	Bos d 4	70	Gly m β-conglycinin
2	Act d 2	37	Bos d 5	71	Gly m glycinin
3	Act d 5	38	Bos d 6	72	Hev b 1
4	Act d 8	39	Bos d 8	73	Hev b 3
5	Aln g 1	40	Bos d lactoferrin	74	Hev b 5
6	Alt a 1	41	Can f 1	75	Hev b 6
7	Alt a 6	42	Can f 2	76	Hev b 8
8	Amb a 1	43	Can f 3	77	Mal d 1
9	Ana c 2	44	Cla h 8	78	Mer a 1
10	Ana o 2	45	Cor a 1.0101	79	Mus m 1
11	Ani s 1	46	Cor a 1.0401	80	Ole e 1
12	Ani s 3	47	Cor a 8	81	Ole e 2
13	Api g 1	48	Cor a 9	82	Par j 2
14	Api m 1	49	Cry j 1	83	Pen a 1
15	Api m 4	50	Cup a 1	84	Pen i 1
16	Ara h 1	51	Cyn d 1	85	Pen m 1
17	Ara h 2	52	Cyp c 1	86	Phl p 1
18	Ara h 3	53	Dau c 1	87	Phl p 11
19	Ara h 8	54	Der f 1	88	Phl p 12
20	Art v 1	55	Der f 2	89	Phl p 2
21	Art v 3	56	Der p 1	90	Phl p 4
22	Asp f 1	57	Der p 10	91	Phl p 5
23	Asp f 2	58	Der p 2	92	Phl p 6
24	Asp f 3	59	Equ c 3	93	Phl p 7
25	Asp f 4	60	Eur m 2	94	Pla a 1
26	Asp f 6	61	Fel d 1	95	Pla a 2
27	Ber e 1	62	Fel d 2	96	Pru p 1
28	Bet v 1	63	Fel d 4	97	Pru p 3
29	Bet v 2	64	Gad c 1	98	Sal k 1
30	Bet v 4	65	Gal d 1	99	Ses i 1
31	Bla g 1	66	Gal d 2	100	Tri a 18
32	Bla g 2	67	Gal d 3	101	Tri a Gliadin
33	Bla g 4	68	Gal d 5	102	Tri a 19.0101
34	Bla g 5	69	Gly m 4	103	Tri a aA_TI
35	Bla g 7				

### IgE-ELISA competition experiments

Enzyme-linked immunosorbent assay (= ELISA) plates (Nunc Maxisorb, Roskilde, Denmark) were coated with *Lolium multiflorum* pollen extract (50 μg/ml) in carbonate buffer and incubated overnight at 4°C. Plates were washed three times with phosphate-buffered saline containing 0.05% Tween (PBS-T), blocked with PBS-T containing 2% BSA (PBS-T-BSA) for 4 hours at room temperature and then washed three times with PBS-T.

For ELISA inhibition experiments, sera from all 78 grass pollen allergic patients were diluted 1:10 in PBS-T-BSA (control) or in PBS-T-BSA containing 100 μg/ml of *Lolium multiflorum* extract, 150 μg/ml of *Phleum pratense* extract, or 20 μg/ml of Phl p 6251 (Biomay, Vienna, Austria). The concentrations of the pollen extracts were determined in pilot experiments to ensure antigen excess. Bet v 1, the major birch pollen allergen, was used at 10 μg/ml for pre-incubation as a control (unrelated allergen). Sera were pre-adsorbed at 4°C overnight, plates were incubated with 100μl/well of the pre-adsorbed sera at 4°C overnight. After five washing steps, bound IgE was detected with horseradish peroxidase (= HRP)-coupled goat anti-human IgE (KPL, Washington, DC, USA) for 1 hour at 37°C, and 1 hour at 4°C., and 2,2'-Azinobis [3-ethylbenzothiazoline-6-sulfonic acid]-diammonium salt (= ABTS) was used as color substrate. The optical density was determined with an ELISA reader. All determinations were carried out in duplicates yielding mean values with less than 10% SD. The percentage of IgE binding inhibition was calculated as follows: [1- (OD_with inhibition_/OD_without inhibition_)] x 100.

### IgE immunoblot inhibition experiments

IgE immunoblot inhibition experiments were performed with sera from patients whose IgE reactivity to *Lolium multiflorum* extract was poorly inhibited with *Phleum pratense* pollen extract. Sera were diluted 1:15 in buffer A and pre-incubated for 4 hours at room temperature with recombinant allergens (mix of rPhl p 1, rPhl p 2, rPhl p5, rPhl p 6 or a mix of purified Phl p 4 and Phl p 12: 20μg/ml each) or with 150μg/ml of *Phleum pratense* or *Lolium multiflorum* pollen extracts, or for control purposes, with buffer A alone. Nitrocellulose-blotted pollen extracts were blocked for 2 hours at room temperature, and after a washing step, incubated with the pre-adsorbed sera overnight at 4°C. After four washing steps, bound IgE antibodies were detected with iodine 125 (^125^I)-labeled anti-human IgE antibodies (RAST; Demeditec Diagnostics, Kiel, Germany), diluted 1:20 in buffer A, and visualized by autoradiography with KODAK X-OMAT films and intensifying screens (Kodak, Heidelberg, Germany) at -70°C.

## Results

### Grass pollen allergic Brazilian patients show an IgE reactivity profile typical for Pooideae sensitization

**Fig 1:**
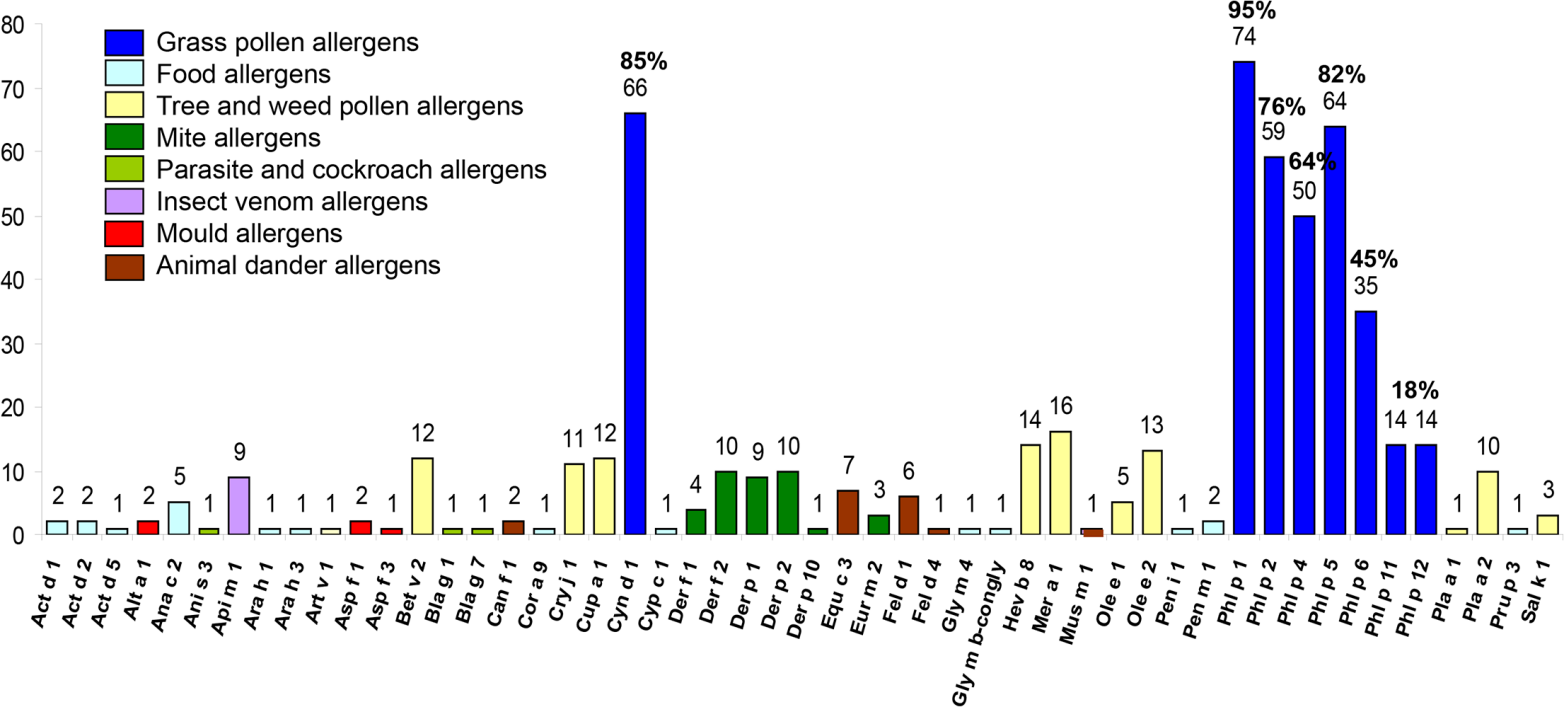
Frequency of allergen recognition by serum IgE from 78 Brazilian grass pollen allergic patients determined by ISAC. Allergens which were tested but not recognized: Act d 8, Aln g 1, Alt a 6, Amb a 1, Ana o 2, Ani s 1, Api m 1, Api m 4, Ara h 2, Ara h 8, Art v 3, Asp f 2, Asp f 4, Asp f 6, Ber e 1, Bet v 1, Bet v 4, Bla g 2, Bla g 4, Bla g 5, Bos d 4, Bos d 5, Bos d 6, Bos d 8, Bos d lactoferrin, Can f 2, Cla h 8, Cor a 1, Cor a 8, Dau c 1, Fel d 2, Gad c 1, Gal d 1, Gal d 2, Gal d 3, Gal d 5, Gly m 5, Gly m 6, Hev b 1, Hev b 3, Hev b 5, Hev b 6, Mal d 1, Par j 2, Pen a 1,Phl p 7, Pru p 1, Ses i 1, Tri a 18, Tri a 19, Tri a aA-TI, Tri a gliadin.

**Fig 2:**
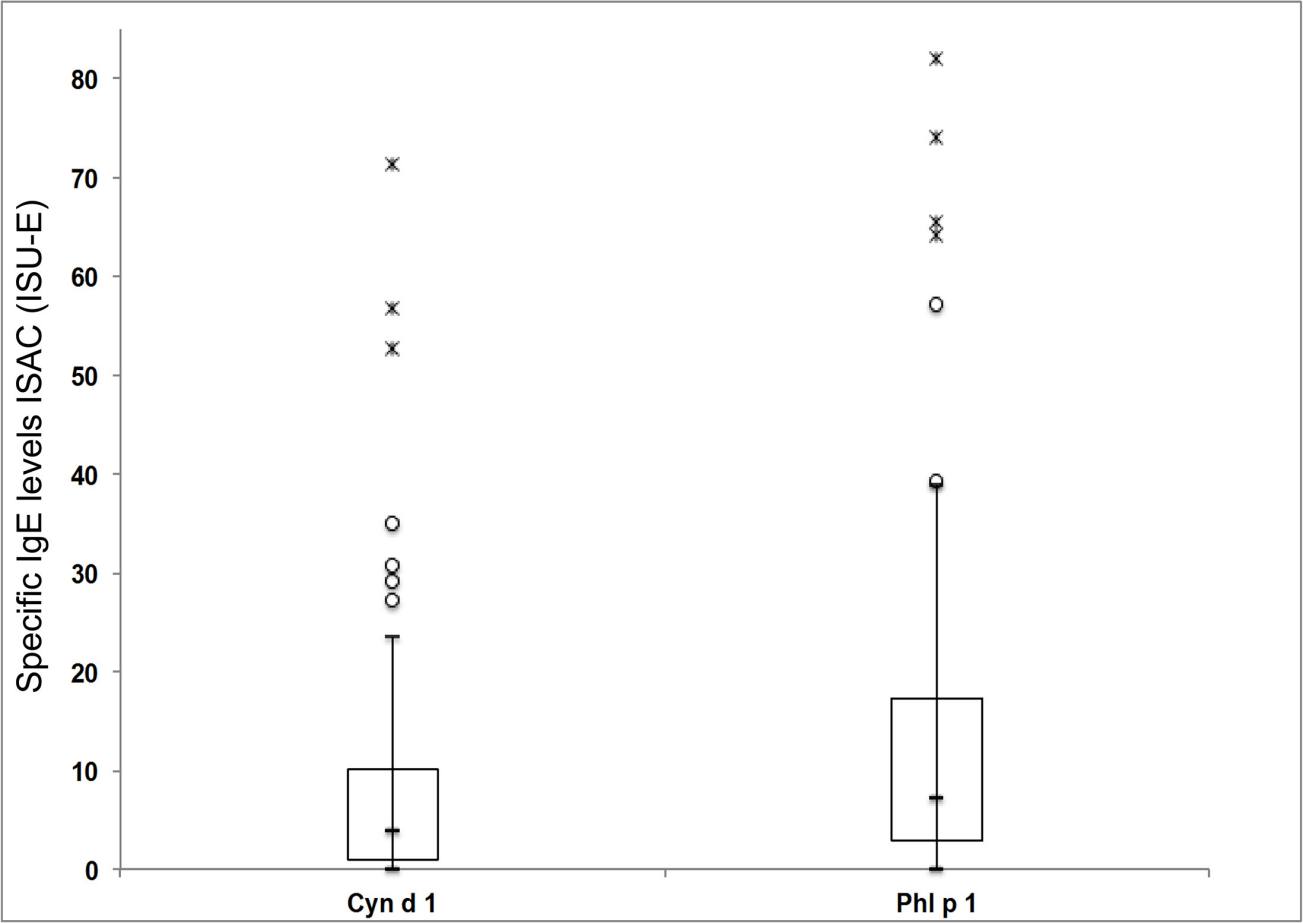
Comparison of the Cyn d 1 and Phl p 1-specific IgE levels in the grass pollen allergic patients. Box plots showing the allergen-specific IgE levels determined by ISAC for Cyn d 1 and Phl p 1. Median values are indicated by a horizontal line in the boxplot, points and asterisks are suspected and complete outliers, respectively. For reasons of better legibility the y-axis stops at 85 ISU-E, thus 1 patient with higher specific IgE (Cyn d 1: outlier at 127,36 ISU-E, Phl p 1: outlier at 152,43 ISU-E) is not shown. Phl p 1-specific IgE levels are significantly higher (p<0.001; paired t-test) than Cyn d 1-specific IgE levels.

Almost all of the grass pollen allergic Brazilian patients (n = 78) suffered from seasonal allergic rhinoconjunctivitis due to grass pollen exposure and each of them showed positive SPT results with *Lolium multiflorum* whereas only two patients presented exclusively with asthma as a symptom. (Table 1) When their sera were analyzed for IgE reactivity to the 103 different micro-arrayed allergens on ISAC we found that they predominantly reacted with grass pollen allergens (Fig 1). The sensitivity of ISAC was high because out of 78 patients who showed positive SPT to *Lolium multiflorum* pollen extract, 77 patients were diagnosed as grass pollen allergic patients using the grass pollen allergens on ISAC. The most frequently recognized allergens were Phl p 1 (95%), Cyn d 1 (85%), Phl p 5 (82%), Phl p 2 (76%), Phl p 4 (64%) and Phl p 6 (45%). Phl p 1 was not only recognized by more patients than Cyn d 1, but also Phl p 1-specific IgE levels were significantly higher than Cyn d 1-specific IgE levels (p<0.001; paired t-test) (Fig 2). Fewer patients had IgE-antibodies to profilin (Phl p 12; 18%) and Phl p 11 (18%) and none was sensitized to the cross-reactive 2 EF-hand calcium binding allergen, Phl p 7. Interestingly, we found that the Brazilian grass pollen allergic patients showed only few concomitant sensitizations to other important allergens, to the extent that they were represented on the ISAC chip. For example, not more than ten of them reacted with the major house dust mite allergens, Der p 1 and Der p 2, respectively. The major cat allergen Fel d 1 and the major dog allergen Can f 1 were recognized by six and by two patients, respectively. Two patients reacted with the major Alternaria allergen Alt a 1 and none of the patients reacted with the major ragweed allergen Amb a 1, the major birch pollen allergen Bet v 1 or the peanut allergen Ara h 2.

### *Phleum pratense* and *Lolium multiflorum* contain a similar spectrum of allergens and show extensive IgE cross-reactivity

**Fig 3:**
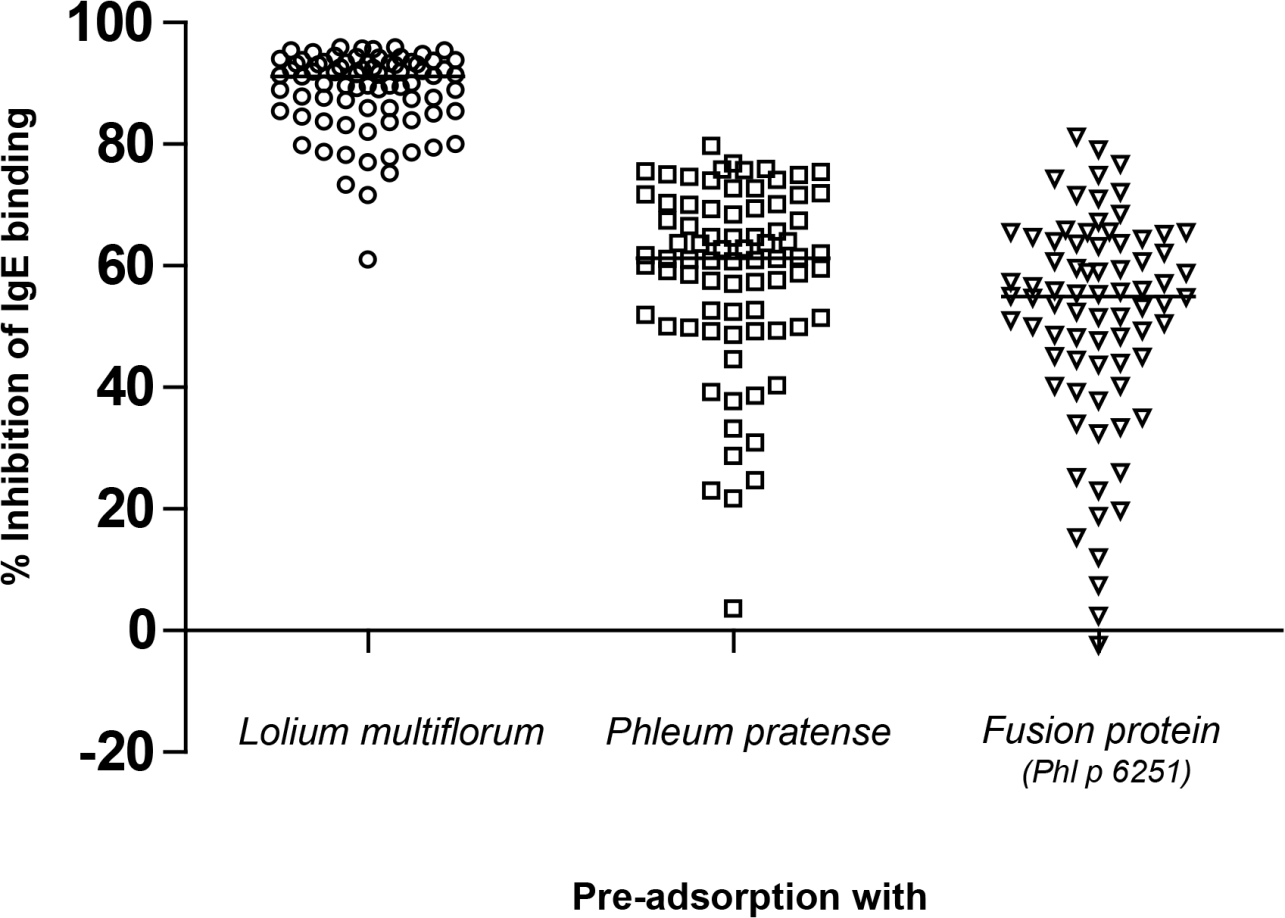
Inhibition of IgE binding to *Lolium multiflorum* extract. Percentages of inhibition of IgE binding (y-axis) after pre-adsorption of sera (n = 78) with *Lolium multiflorum* extract (left panel), *Phleum pratense* extract (middle panel) or the rPhl p 6251 fusion protein (right panel, x-axis). Data are represented in scatter plots, the lines inside the plots indicate the median.

We analyzed the allergen profile of *Lolium multiflorum*, the predominant grass in the area where the patients lived. Using a panel of allergen-specific probes we detected group 1, 2, 4, 5, 7, 12 and 13 allergens but not group 6 allergens in *Lolium multiflorum* extract (data not shown). To determine the level of cross-reactivity of IgE epitopes between allergens in *Lolium multiflorum* and *Phleum pratense* pollen extracts, ELISA inhibition experiments with *Lolium multiflorum* on the solid phase were performed. The level of auto-inhibition (i.e., pre-adsorption of sera with *Lolium multiflorum* extract) was 89±7% on an average (Fig 3, left panel). Incubation of sera with *Phleum pratense* extract inhibited IgE binding to *Lolium multiflorum* extract by 59±15% on an average (Fig 3, middle panel). When the sera were pre-adsorbed with the rPhl p 6251 fusion protein consisting of Phl p 1, Phl p 2, Phl p 5 and Phl p 6, the mean inhibition was 51±18% (Fig 3, right panel). The level of cross-reactivity between *Phleum pratense* and *Lolium multiflorum* was 66.3% and 57% of *Lolium multiflorum* IgE epitopes were represented on the single fusion protein, rPhl p 6251.

### Few patients show IgE reactivity to moieties in *Lolium multiflorum* pollen extracts which do not cross-react with *Phleum pratense*

**Fig 4:**
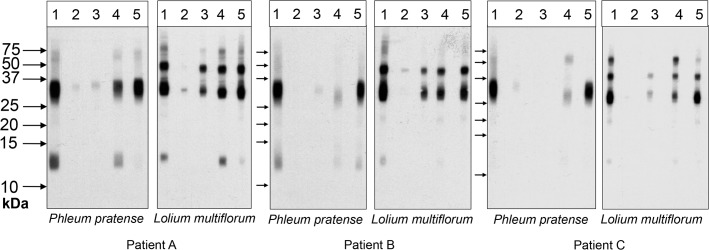
IgE immunoblot inhibitions. Sera from three patients (A, B, C) were pre-adsorbed with buffer (1), *Lolium multiflorum* pollen extract (2), *Phleum pratense* pollen extract (3), a mix of rPhl p 1, rPhl p 2, rPhl p 5 and rPhl p 6 allergens (4), or with a mix of purified nPhl p 4 and nPhl p 12 allergens (5) and reacted with nitrocellulose-blotted *Lolium multiflorum* or *Phleum pratense* pollen extract. Molecular weights are displayed on the left side.

IgE reactivity in six of the 78 grass pollen allergic patients to *Lolium multiflorum*, representing 7.7% of the study population, was poorly inhibited with *Phleum pratense* extract (<10%). Representative IgE immunoblot inhibition experiments from three patients are shown in Fig 4. Immunoblot inhibition results revealed two bands of approximately 30 and 50 kDa in *Lolium multiflorum* extracts which were only partly cross-reactive (Fig 4).

## Discussion

In the present study we analyzed a population of grass pollen allergic patients from Southern Brazil, who are exposed to a variety of grasses, which differ from those usually encountered in Europe or North America. These patients were exposed to grasses belonging to the Pooideae mainly represented by *Lolium multiflorum* but also to members of the subfamily Chloridoideae such as Bermuda grass. The spectrum of allergens in these two subfamilies differs fundamentally. Chloridoideae mainly contain group 1, 4 and 13 allergens but lack group 2, 5 and 6 allergens, which are present in the Pooideae. [14] Group 6 allergens have been found mainly in timothy grass and Kentucky Bluegrass but are absent from many other members of the Pooideae. [23] The determination of the IgE reactivity profiles in the Brazilian grass pollen allergic patients with ISAC, a chip containing more than 100 micro-arrayed allergens revealed that Phl p 1 (95%) and Phl p 5 (82%) the group 1 and 5 allergens from timothy grass, Cyn d 1 (85%) from Bermuda grass and the group 2 allergen from timothy grass, Phl p 2 (76%) were the most frequently recognized grass pollen allergens. This IgE reactivity profile is highly indicative of a sensitization to Pooideae, which is in accordance with the fact that *Lolium multiflorum* a member of the Pooideae is abundant in this area. A primary sensitization to Bermuda grass and tropical grasses is unlikely because Phl p 1 was more frequently and more strongly recognized than the Bermuda grass allergen, Cyn d 1. However, 45% of the patients reacted with Phl p 6, a group 6 allergen which so far has not been detected in *Lolium multiflorum*. In fact, using group 6 allergen-specific antibodies, we showed that no group 6 allergen was present in *Lolium multiflorum* pollen extract. The recognition of Phl p 6 in the Brazilian population is therefore most likely due to cross-reactivity with group 5 allergens: In fact it has been shown, that there is a high degree of sequence homology between group 6 and the N-terminal portions of the group 5 allergen. [23,24] The observed molecular IgE reactivity profile therefore strongly suggests that primary sensitization in the investigated population occurred to *Lolium multiflorum*. Despite this, all but one of the 78 grass pollen allergic patients could be diagnosed by IgE serology using the micro-arrayed timothy grass pollen allergens, indicating that this panel is useful for diagnosis of grass pollen allergy in the Brazilian population.

Another interesting side aspect of the analysis of IgE reactivity profiles in the Brazilian grass pollen population was the finding that these patients showed only few concomitant sensitizations to other important respiratory allergen sources such as pets, moulds, weeds and trees as well as food allergen sources when analysed using the ISAC chip and in this respect seem to differ substantially from grass pollen allergic patients in Europe and America [25]. This finding is interesting because the grass pollen allergic patients included in this study had not been pre-selected to be mono-sensitized to grass pollen but were consecutively recruited in the clinic. The lack of some of the above mentioned sensitizations may be explained by the absence of certain allergen sources such as birch, ragweed and mugwort.

We also found a smaller percentage of house dust mite sensitization in our study population using the ISAC assay: Der p 1: 9/78 = 11.5%; Der p 2: 10/78 = 12,8%; Der f 1: 4/78 = 5.1%; Der f 2: 10/76 = 13,1% than was found in the initially done SPTs, where the patients showed the following results: *Dermatophagoides pteronyssinus* (45% positive), *Dermatophagoides farinae* (27% positive) and *Blomia tropicalis* (29% positive). Given the well-known high prevalence of sensitization to house dust mite in Brazil, especially in the South-East region [26,27], the observed discrepancy between SPT results and ISAC assay results as far as mite allergens are concerned may be due to the fact that the study population is sensitized to other house dust mite allergens not yet present on the ISAC chip such as Der p 23, and/or allergens from tropical mites, which are also not yet fully represented on the chip [28–30].

In terms of clinical symptoms only very few patients presented with mild to moderate asthma during the pollen season (September-December). However, this is in keeping with the general prevalence of asthma of about 10% in this region.

When we measured the level of cross-reactivity between *Lolium multiflorum* and timothy grass pollen extract in our population in ELISA experiments, we found a considerable level of IgE cross-reactivity (66.3%) between the two extracts. Even pre-incubation with a single recombinant fusion protein, which carries IgE epitopes from Phl p 1, Phl p 2, Phl p 5 and Phl p 6, led to substantial inhibition of IgE binding to *Lolium multiflorum* extract.

To the best of our knowledge our study represents the first published dissection of allergen sensitization to multiple micro-arrayed allergens in a population from South America. One limitation of our study is that we could analyse only patients from one area in Brazil but nevertheless our results indicate that recombinant Phleum pratense allergens can be used to diagnose grass pollen allergy in this population living in subtropical high altitude zones of Brazil, even though Lolium and not Phleum represents the most abundant grass pollen species in this area.

In fact, it has been shown that a mix of recombinant timothy grass pollen allergens (i.e., rPhl p 1, 2, 5 and 6) could be used for successful immunotherapy in a European population [16]. Our study indicates that at least in the tested population a similar mix may be used for specific immunotherapy and thus provides information regarding the composition of grass pollen allergy vaccines required for the investigated population.
